# Supplementary material for: CeO2-rGO Composites for Photocatalytic H2 Evolution by Glycerol Photoreforming
Source: Materials (Basel). 2023 Jan 12;16(2):747. doi: 10.3390/ma16020747 (PMC9863801; doi:10.3390/ma16020747)
Supplement: Supplementary file 1 [file materials-16-00747-s001.zip › materials-2146325-supplementary.pdf]

CeO_2-rGO Composites for Photocatalytic H_2 Evolution by Glycerol Photoreforming

Stefano Andrea Balsamo[1*], Eleonora La Greca[2], Marta Calà Pizzapilo[1], Salvatore Sciré[1] and Roberto Fiorenza[1]

1 Department of Chemical Sciences, University of Catania, Viale A. Doria 6, 95125 Catania, Italy; marta.cala20@gmail.com (M.C.P.); sscire@unict.it (S.S.); rfiorenza@unict.it (R.F.)

2 Institute for The Study of Nanostructured Materials (ISMN)-CNR, Via Ugo La Malfa 153, 90146 Palermo, Italy; eleonora.lagreca@hotmail.it

* Correspondence: stefano.balsamo@phd.unict.it; Tel.: +39-333-821-5178

SUPPLEMENTARY MATERIALS

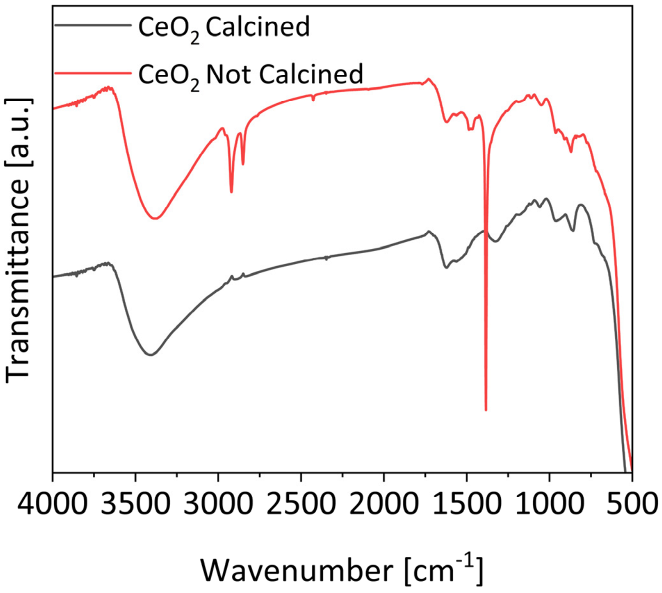

Figure S1. FTIR spectra of the CeO_2 samples before and after the calcination.

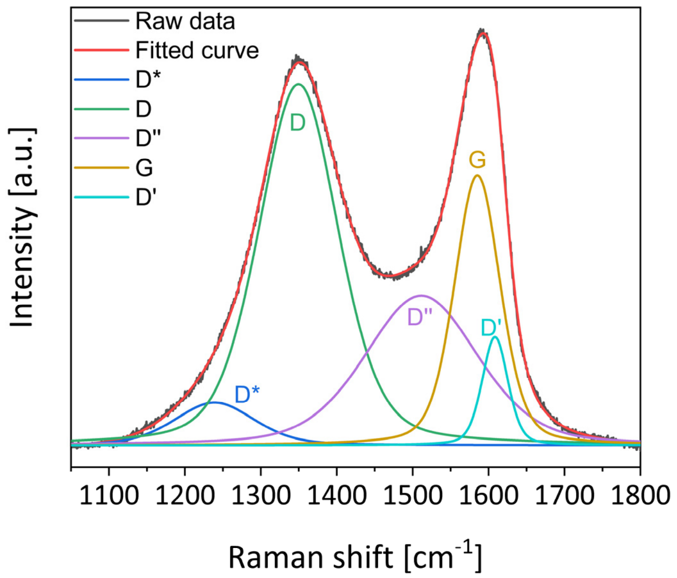

Figure S2. Raman signals deconvolution of D and G bands in their five different components for the bare GO as representative sample.

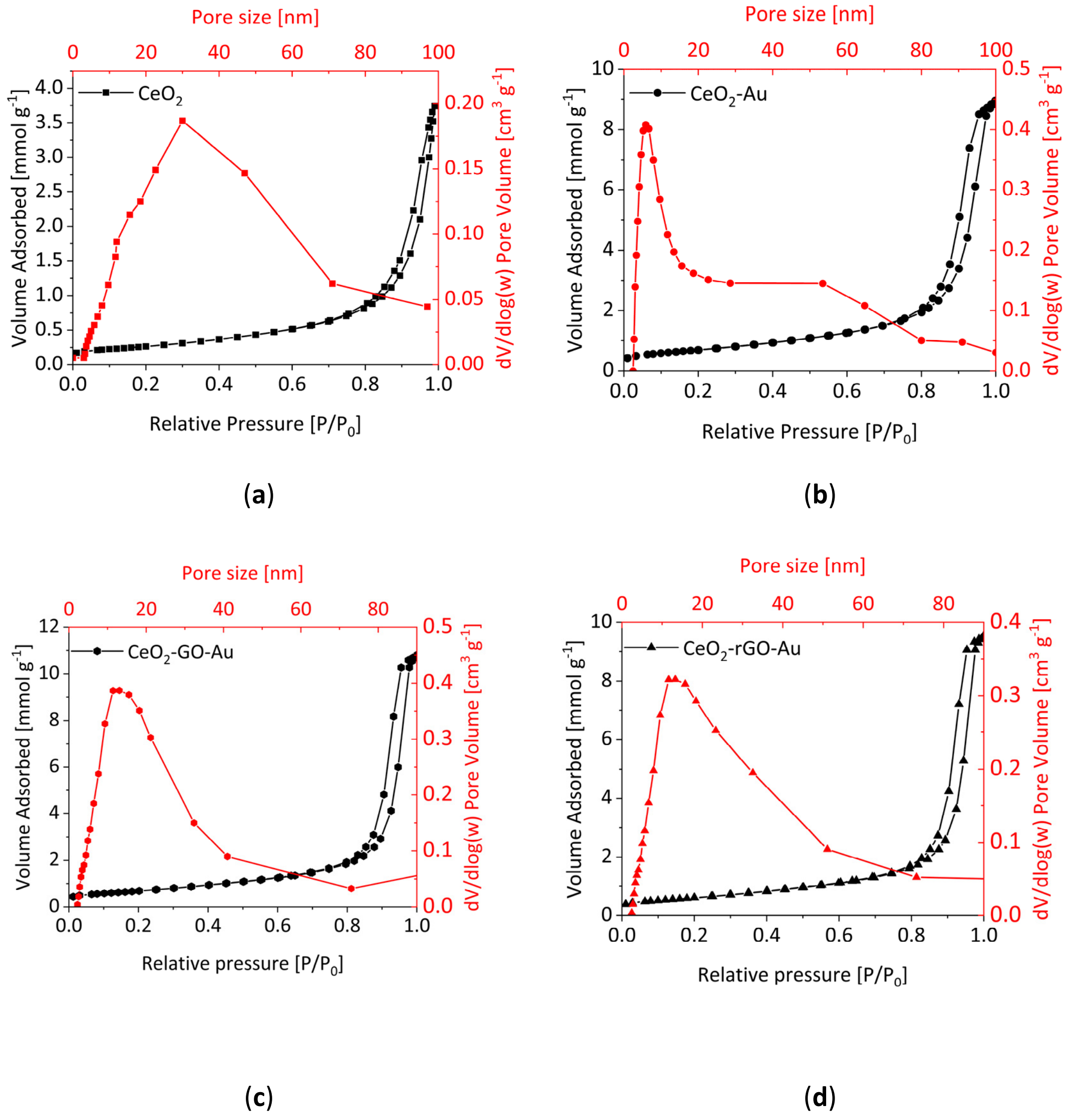

Figure S3. N_2 adsorption-desorption isotherms and pore size distribution of: (a) CeO_2, (b) CeO_2-Au, (c) CeO_2-GO-Au and (d) CeO_2-rGO-Au.

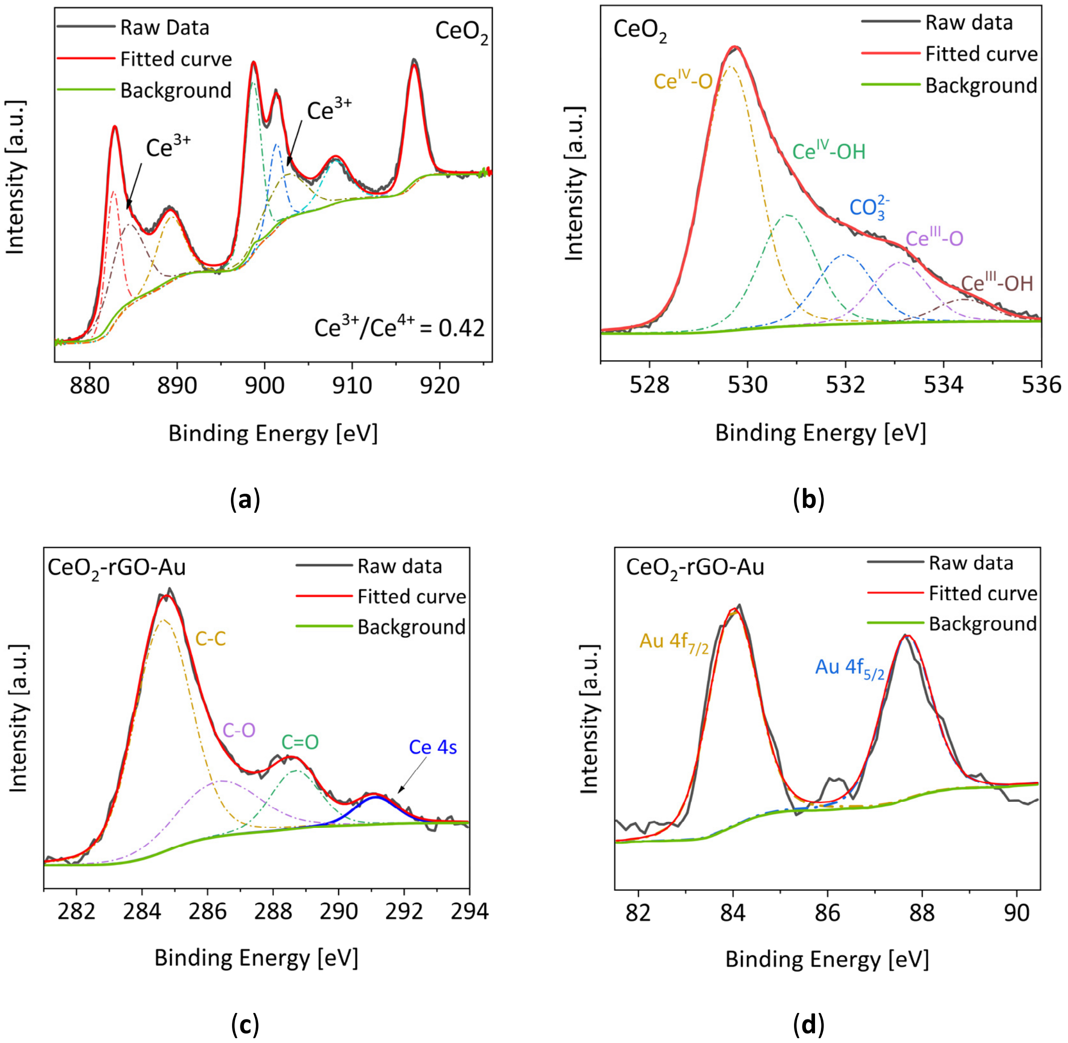

Figure S4. XPS spectra of: (a) Ce 3d of bare CeO_2, (b) O 1s of bare CeO_2, (c) C 1s of CeO_2-rGO-Au and (d) Au 4f of CeO_2-rGO-Au.

Table S1 XPS signals deconvolution. The values are in binding energy (eV).

Sample	Ce 3d (eV)	O 1s (eV)	Au 4f (eV)
CeO_2	881.7, 883.5, 888.3, 897.6, 900.3, 901.8, 907.1, 916.0	529.7, 530.8, 532.8, 533.1, 534.4	/
CeO_2-GO	881.9, 883.7, 888.3, 897.7, 900.2, 902.0, 907.3, 916.3	529.6, 530.7, 532.5, 532.9, 534.2	/
CeO_2-rGO	881.5, 883.4, 888.2, 897.6, 900.2, 901.7, 906.9, 915.9	529.8, 531.0, 532.7, 533.0, 534.4	/
CeO_2-GO-Au	881.7, 883.4, 888.2, 897.6, 900.2, 901.9, 907.1, 916.0	529.7, 530.7, 532.6, 533.2, 534.3	84.0, 87.5
CeO_2-rGO-Au	881.6, 883.4, 888.2, 897.6, 900.1, 901.7, 907.0, 916.0	529.7, 530.8, 532.7, 533.0, 534.3	84.0, 87.5
